# Bayesian optimisation of a bio-based biodegradable polyester thermoset

**DOI:** 10.1039/d6su00235h

**Published:** 2026-09-29

**Authors:** Thomas Højlund-Dodd, James A. Elliott

**Affiliations:** a Department of Materials Science and Metallurgy, University of Cambridge Charles Babbage Road Cambridge CB3 0FS UK jae1001@cam.ac.uk

## Abstract

Glass fibre-reinforced polymer composites are widely used in the wind turbine manufacturing industry, and are commonly manufactured using general-purpose unsaturated polyester resin thermosets. However, these resins are obtained from oil-derived aromatic monomers making them both unsustainably sourced and difficult to recycle. Bio-based and biodegradable alternatives exist but have not yet seen widespread commercial success due to their relatively high cost and poor mechanical properties. This paper seeks to address the question of whether bio-based branching thermoset polyesters can be optimised using Bayesian optimisation such that properties correlated with mechanical performance, including extent of polymerisation, might be improved upon. This is achieved by selecting a bio-based and biodegradable polymer (poly(glycerol citrate itaconate)), identifying a desirable objective variable (mass loss under esterification as proxy for extent of polymerisation) and some tunable predictor variables (monomeric stoichiometry), and then applying sequential Bayesian techniques to uncover well-performing samples in an experimentally efficient manner. A Bayesian technique employing the probability of improvement acquisition function discovered the experimental global optimum stoichiometry producing a mass loss of 20.8% in curing polyester samples, which represents 72% of the theoretical maximum, indicating a high extent of polymerisation. Bayesian techniques employing both expected improvement and probability of improvement acquisition functions were simulated 10 000 times across five different emulations of the practical experiment's parameter space. For one particular method (GP-Mat-05), the estimated probabilities of reaching the defined region of global optimality were 65.0% and 23.8%, respectively. We conclude that Bayesian optimisation techniques represent a practically deployable and sample-efficient means by which desirable properties of bio-based thermosets may be improved.

## Abstract 2

Bayesian optimisation efficiently navigated a slow-curing polyester formulation space and identified improved compositions. Across five emulated response surfaces, expected improvement most consistently reached high-performing regions.

## Graphical abstract

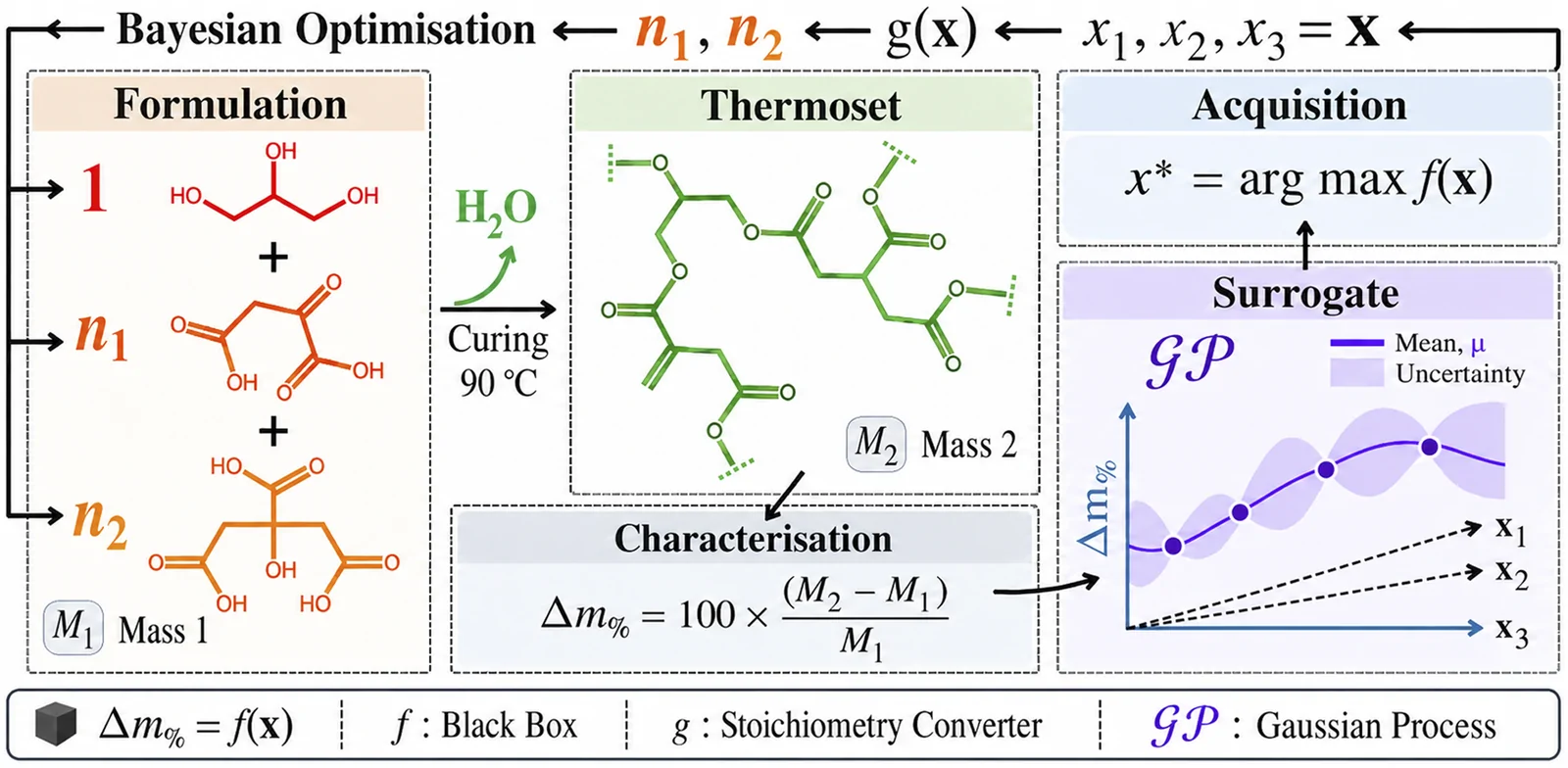

## 1 Introduction

The early 21st century has seen continued growth in both the global human population (from 6.17 to 8.09 billion people) and total energy supply required to meet its needs (from 416 to 634 EJ) between 2000 and 2023.^1–3^ In spite of continued strong reliance upon coal, natural gas, and oil in order to satisfy this demand, concerns regarding global climate and the finite nature of fossil reserves have galvanised investment in alternative renewable power systems.^3,4^ Of these renewables, wind power saw the greatest growth in capacity to generate electricity (+2.33 PWh) ahead of both hydroelectric (+1.69 PWh) and solar photovoltaics (+1.60 PWh).^5^ Wind turbine blades are commonly manufactured from a composite comprised of thermosetting polymer matrix and reinforcing material; these strong thermoset matrices are chemically crosslinked which makes them difficult to reprocess or remould.^6^ Given the relatively low rate of recycling and a common rate of replacement (*ca.* every fifteen years in China^7^) the industry generates a large volume of practically unrecyclable thermoset waste which contravenes the UN's 12th SDG to “ensure sustainable consumption and production”.^8^ In addressing the industry's sustainability shortcoming identified above, the literature is divided into two groups: firstly, papers which focus upon improving the recyclability of contemporary materials, whether by thermal, chemical, mechanical or high-voltage fragmentation methods;^9–11^ and secondly, papers aimed at the development of properties in future replacement materials that would facilitate recycling,^12^ such as introduction of chemically cleavable bonds into the thermoset polymers used.^13^ The motivation for the present work was to ascertain whether Bayesian optimisation could be used to sample efficiently improve the extent of polymerisation in a novel biodegradable bio-based thermoset.

The composite matrix used for wind turbine blades is comprised of a moulding thermoset, with the most commonly manufactured pre-thermoset form being general-purpose unsaturated polyester resin (GP-UPR).^14^ These GP-UPRs are based on a mixture of a reactive diluent (*e.g.* styrene) and a linear aromatic unsaturated polyester (*e.g.* poly(propylene maleate phthalate)), which are cured chemically or thermally to generate the final thermosetting matrix.^15^ Although polyesters are known to exhibit limited biodegradability (due to their labile ester bonds being prone to both biotic and abiotic hydrolysis^16^) it is the case that polyesters with aromatic moieties are more resistant than aliphatic counterparts to such degradation.^17^ Consequently, thermosets derived from GP-UPRs synthesised from the aromatic monomer phthalic acid^15^ can be reasonably expected to exhibit a high resistance to biodegradation; which was part of the reason for their original choice as a material. An archetypal example of an aliphatic polyester would be poly(glycerol sebacate), which uses the monomers glycerol (triol) and sebacic acid (dicarboxylic acid) to form a branching polyester thermoset on thermal curing *via* polyesterification.^18^ Given their natural biodegradability, in the present work we chose to focus upon aliphatic polyesters.

Another concern associated with GP-UPRs with respect to the UN's 12th SDG is that of a material's provenance. If the underlying chemistry of a polymer is petrochemical in origin, then any carbon liberated from the material at end-of-life (whether by combustion for energy or biodegradation in landfill) will release fossil carbon, contributing *via* an anthropogenic route towards global climate change. Since GP-UPRs are synthesised from the oil-derived feedstock monomers propylene glycol, maleic acid, and phthalic acid, the resultant thermosets contain endogenic (fossil-derived) carbon.^19^ In the present work we aim to avoid these feedstocks and focussed instead upon a bio-derived alternative, with inspiration drawn from a chemical system presented in the literature by Alberts, where a combination of glycerol and citric acid were cured to form a bio-based branched aliphatic polyester thermoset.^20^ We build upon this research by adopting the chemical system and adapting it to include a third monomer, itaconic acid, which provides a second carboxylic acid with a lower degree of functionality (dicarboxylic acid); this second lower-functionality molecule was expected to encourage the emergence of alternative thermoset networks. Notably, Alberts found that physical properties of the end thermoset poly(glycerol citrate) could be tuned by modifying the monomeric stoichiometries of the prepolymer resin synthesised.^20^ Given this, we identified mass loss as a variable of interest during the synthesis of poly(glycerol citrate itaconate) as it is a proxy for extent of polymerisation, and sought to optimise this by tuning the stoichiometries of the prepolymer resins mixed. Th extent of polymerisation could be an indicator of the crosslink density of these samples; a characteristic, which, if maximised, yields improvements in mechanical properties such as ultimate tensile strength, Young's modulus, and glass transition temperature.^21^ Note that the link between sample mass loss and polymerised content is predicated on various assumptions: negligible volatilisation of monomers during curing, a lack of side-reactions between monomers, and a negligible mass of water retained within the final cured matrices of samples. The present work also details a standalone experiment which links this mass loss proxy variable with tetrahydrofuran-insoluble fraction in cured thermoset samples.

Poly(glycerol citrate itaconate) is formed from its monomers by polyesterification, a process which is dependent upon variables such as temperature, stoichiometric ratios, and additive presence.^20^ Additionally, reaction times for rigid solid thermoset materials are on the order of days when uncatalysed^22^ (catalysis commonly involves strong fuming acids which can corrode equipment^23^). Given the high dimensionality and temporally expensive function evaluation exhibited by this family of polyesters, a sample efficient global optimisation technique, namely Bayesian optimisation, was selected to tackle this problem. Alternative global optimisation techniques were considered but ruled out; for example, the hill-climbing class gradient descent algorithm is reliant on derivatives of the objective function which would have been uncertain given the noisy function evaluations associated with physical systems as opposed to simulated ones.^24^ The population class evolutionary and particle swarm optimisation algorithms were suitable but avoided in favour of the more sample efficient surrogate class of algorithms. Surrogate class algorithms are variously formulated, but the three major forms are distinguished by the surrogate models they leverage: polynomial response surface model, kriging (also known as Gaussian process regression) and radial basis functions.^25^ Kriging is preferable to response surface modelling since it makes no assumption of polynomial order, avoiding *a priori* bias in the surrogate models generated. Furthermore, radial basis function-based surrogate models provide no estimates of uncertainty around the fitted function; functions and associated uncertainties derived using kriging provide the means by which Bayesian optimisation effectively balances exploitation and exploration of the parameter space. Hence, for the development of a part-computational and part-laboratory based workflow for the optimisation of mass loss in poly(glycerol citrate itaconate) we decided to use Bayesian optimisation predicated on kriging.

Originally coined in the 1970s by Mockus *et al.*,^26^ Bayesian optimisation was later developed further in the 1990s by Jones *et al.* into a technique called the efficient global optimisation algorithm.^27^ This name and technique was thereafter adopted and popularised by the DoE community. More recently, the original name has seen a revival as the technique and several adaptations have been re-discovered and implemented in a range of contemporary communities including robotics,^28^ bioengineering,^29^ and machine learning.^30^ In materials science, Bayesian optimisation has been used to predict crystalline structures,^31^ carry out sample-efficient optimisation of density functional theory simulations,^32^ and multi-objective optimisation of stability and ion-conductivity in materials.^33^

This paper begins by detailing the polymeric system considered, experimental means by which samples were manufactured and characterised, as well as the means by which a suitable parameter space was devised. Hereafter, we detail the algorithmic mechanism behind the Bayesian techniques used before further exploring the initial one-shot quasirandom sampling strategy, sequential Bayesian acquisition functions, and one-shot benchmark sampling routines applied during the course of this work. The methods section concludes with a brief overview of how the physical system was computationally emulated such that Bayesian techniques could be simulated repeatedly to gain statistical insights. The results section is split between elucidation of results associated with the practical optimisation of the physical system, and those which derived from repeated simulation of optimisation routines carried out upon the emulated system. The paper concludes that the Bayesian sequential routines succeeded in improving the mass loss metric from 48% to 72% of the theoretical limit. In simulations using the GP-Mat-05 emulator, the probability of improvement and expected improvement acquisition functions respectively exhibited probabilities of 23.8% and 65.0%, respectively, of discovering values approaching the emulated global optimum, far surpassing the various benchmark techniques.

## 2 Method

An integrated experimental-computational methodology was developed to optimise the properties of the poly(glycerol citrate itaconate) thermoset, supported by custom software enabling human-in-the-loop optimisation in a laboratory setting. Programs were written in the Python programming language and developed into modules, whereafter these were united into a single package, “BO4BT”, available from a GitHub repository (https://github.com/ThomasDodd97/BO4BT) alongside its dependencies. A .yaml configuration file is also provided which allows an identical Conda environment to be set up, allowing a user to run this code.^34^

### 2.1 Chemical background

The condensation reaction occurring between carboxyl and hydroxyl groups is an esterification reaction:

1 RCOOH + HOR′ → RCOOR′ + HOH

As opposed to the simple esterification shown in eqn (1), the desired polyester thermoset, poly(glycerol citrate itaconate), is the product of polyesterification between multi-functional monomers.

Polyesterification is a form of polycondensation reaction which takes place as follows: an initial ester is formed from two difunctional monomers, where one must at least be a diol and the other a diacid (alternatively, each may possess a carboxyl and a hydroxyl group), producing a molecule of water and an ester with two spare functional groups-these groups can subsequently react with more difunctional monomers causing polymerisation into a linear polyester (see Fig. 1). When monomers with a functionality greater than two are introduced into a polyesterification reaction, then side chains can form along the primary backbone of the growing polyester, which causes the development of a branching polymeric structure (see Fig. 2). When a side chain reaches another large growing polyester molecule and reacts with it a crosslink is said to have developed, and when widespread crosslinking occurs generating a 3D network of polyester molecules a thermoset polyester has been created. Fig. 2 shows how the monomers employed in the synthesis of poly(glycerol citrate itaconate) give rise to this branching thermoset structure.

**Fig. 1:**
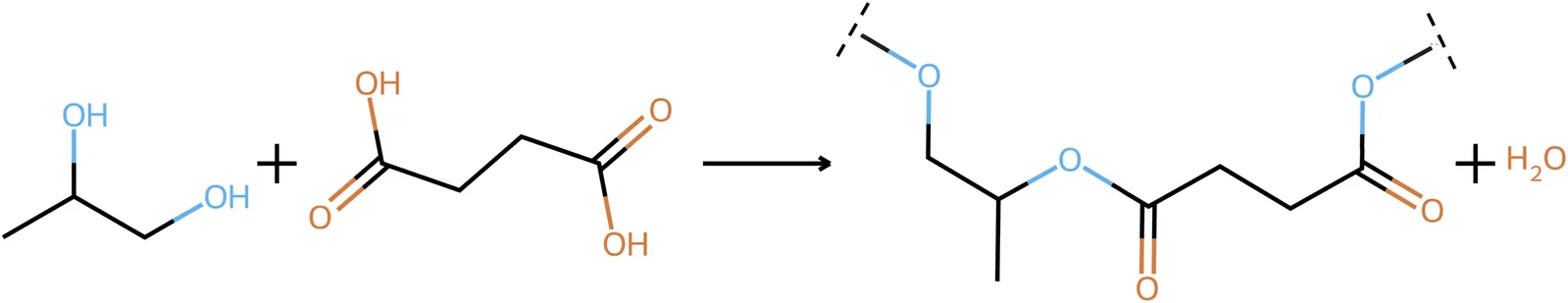
Archetypical polyesterification between a diol (propylene glycol) and a diacid (succinic acid) forming a linear polyester. Functional groups participating in the reaction are highlighted: hydroxyl groups blue, and carboxyl groups orange. In the products, ester groups are blue and carbonyl groups and water are also orange in accordance with provenance.

**Fig. 2:**
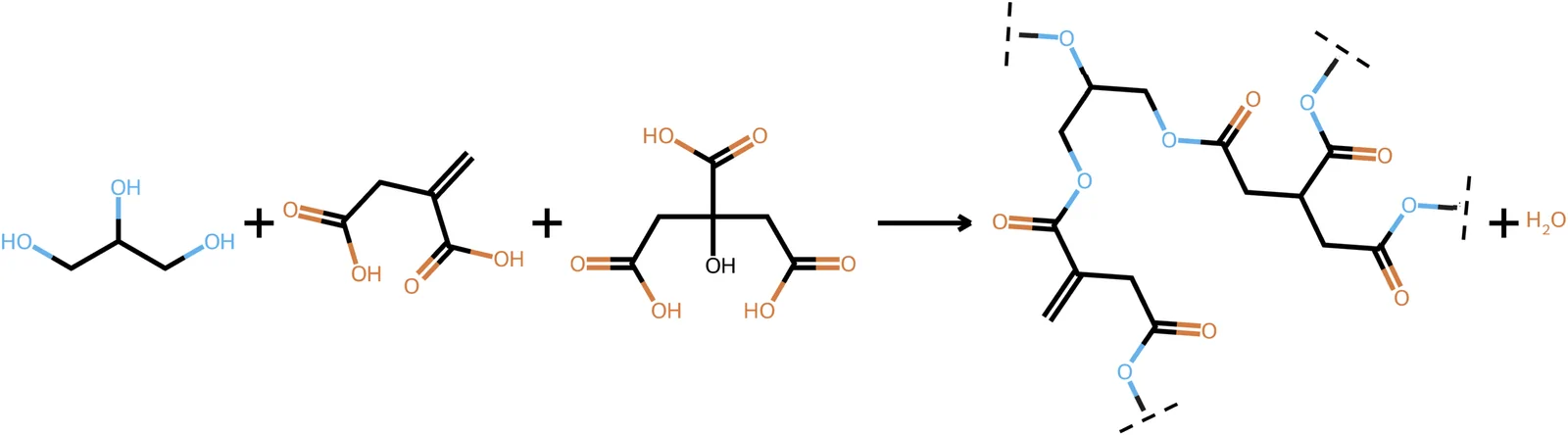
Polyesterification between glycerol, citric acid, and itaconic acid forming poly(glycerol citrate itaconate). Functional groups participating in the reaction are highlighted: hydroxyl groups blue, and carboxyl groups orange. In the products, ester groups are blue and carbonyl groups and water are orange in accordance with provenance.

Importantly, during the polyesterification process water is generated as the main by-product, and hence it is assumed that virtually all mass loss between prepolymer and final cured thermoset would be associated with water evaporation. Given that each molecule of water produced corresponds to an ester bond formed, mass loss can be said to be a good proxy for the extent of polymerisation within a sample.^22^ Thus, this research identifies mass loss as a metric of interest and sought to maximise this during optimisation of poly(glycerol citrate itaconate) synthesis: increasing the extent of polymerisation and thereby improving the degree of crosslinking and associated strength of these bio-based thermosets.

### 2.2 Experimental method

Synthesis of the poly(glycerol citrate itaconate) thermoset was achieved *via* an uncatalysed polyesterification reaction (see Fig. 2). Acid catalysis was avoided to reduce corrosion damage to equipment, this ultimately reduced the rate at which this reaction could be carried out but did not prevent polycondensation.^32^ The monomers glycerol (99+ %), citric acid (99.6%), and itaconic acid (99+ %) used were supplied by Fisher Scientific International and manufactured by Acros Organics.

Thermoset synthesis was carried out in two steps: firstly, a homogenous prepolymer resin was prepared by stirring solid monomers (acids) together with liquid monomer (glycerol) atop a hotplate and raising the temperature to a maximum of 140 °C until the solids were completely dissolved. The temperature of 140 °C was chosen to accelerate dissolution without encouraging decarboxylation of the citric acid to itaconic acid.^20^ Secondly, prepolymer was cured into the desired thermoset by transferral to a silicone mould before being exposed to elevated temperatures (90 °C) for an extended period (up to a week) within a drying oven.

Preliminary experimentation showed that temperatures exceeding 100 °C, caused boiling of byproduct water within samples. Gaseous water from boiling was entrapped within the curing polyester creating bubbles within the final structure, resulting in unpredictable densities of the final material. Given the unpredictable nature of samples generated in this fashion, drying oven temperatures were held below 90 °C to avoid this uncontrollable effect upon the experiments.

As defined by eqn (2), determination of mass loss associated with the synthesis of a thermoset sample (Δ*m*) was obtained by measuring the difference between both the initial mass of prepolymer resin (*m*_p_) and the final mass of cured thermoset (*m*_t_).

2 Δ*m* = *m*_p_ − *m*_t_

Eqn (3) defines sample mass loss (Δ*m*) normalised into a percentage format (Δ*m*%), this allowed inter-comparison since individual samples had slightly varying initial prepolymer resin masses (*m*_p_).3
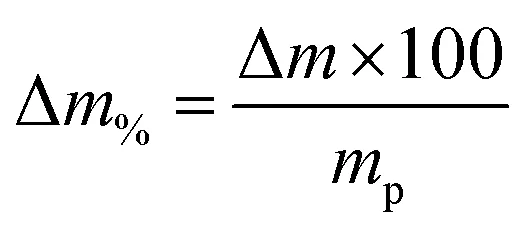


Taking into account an initial prepolymer mass of 3 g, a mass loss range spanning 0.2–0.6 g, and a mass balance repeatability of 0.01 g, we could estimate the uncertainty with respect to Δ*m*_%_ using a Monte Carlo sampling procedure and some assumed distributions - this equated to 0.44% points with 1*σ* = 0.01% points (see SI in Section 8.1). Residence time within the drying oven for a sample was controlled by periodic measurement of sample mass: mass was plotted against time, an exponential decay curve fitted, and the sample removed from the oven once a pre-determined rate of mass loss was reached (this equated to around −0.028% h^−1^ of the initial mass).

To provide independent evidence of network formation occurring as a function of the sample mass loss proxy variable, a practical experiment was undertaken wherein five thermoset sample formulations were tested for tetrahydrofuran-insoluble fraction, with the method adapted from Susa *et al.* (2024).^35^ The THF-insoluble fraction was ascertained by firstly taking an initial mass measurement *m*_initial_ (in grams) of a cured thermoset sample. Secondly, this sample was exposed to room-temperature THF for a period of 12 hours under agitation, before being dried at 40 °C until a constant mass reading was obtained *m*_dried_ (in grams). Thirdly, eqn (4) was used to ascertain a THF-insoluble fraction value *g* (in %) for the thermoset sample considered.4
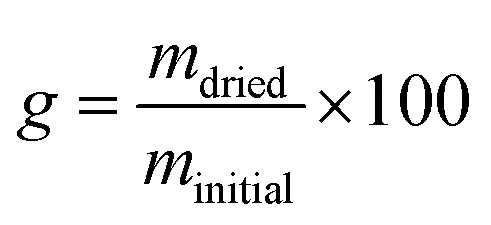


For the purposes of this particular experiment all measurements were undertaken using a microbalance with a readability of 0.01 mg, Monte Carlo investigation of error propagation found the errors associated with both *g* and Δ*m*_%_ to therewith to be negligible (around 0.0004% for both metrics).

Having prepared and cured five different formulations from across the parameter space investigated, these samples were exposed to the THF as described. Three of these remained sufficiently intact for quantitative recovery of the insoluble material, across these, sample mass loss closely followed gel fraction increase, as presented in Fig. 3. Two samples disintegrated during tTHF exposure and could not be quantitatively recovered, and were therewith treated as failed measurements rather than zero insoluble fraction. Despite the sample number limitation, this series of measurements supports the use of sample mass loss under curing as an empirical indicator of network formation.

**Fig. 3:**
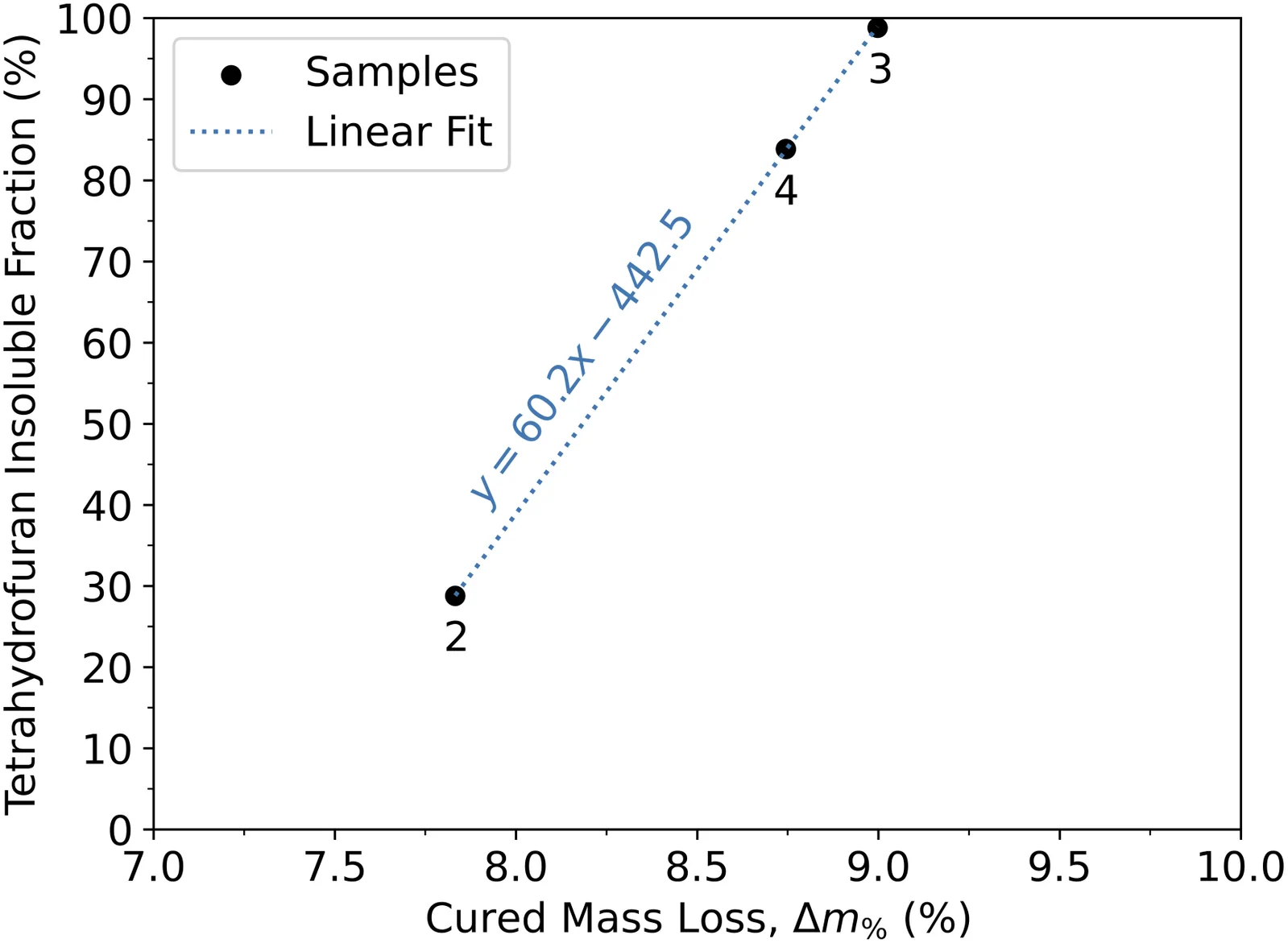
Overview of the relationship between the mass loss proxy variable and THF-insoluble fraction associated with a given formulation. Numbering indicates the 3 samples which were recoverable and characterised during the course of experimentation.

### 2.3 Optimisation framing

Having selected percentage mass loss within a thermoset sample (Δ*m*_%_) as a suitable target variable for optimisation, predictor variables were defined and constrained creating a suitable parameter space describing a full range of prepolymer monomeric compositions.

The polyesterification of monomers to polymer seen in Fig. 2 is presented in eqn (5) as a parameterised stoichiometric equation linking monomeric reagents glycerol (G), itaconic acid (It), and citric acid (Ci) with the product polymer poly(glycerol citrate itaconate) (P(GCiIt)) and byproduct water. Eqn (5) introduces three stoichiometric variables representing the moles of glycerol (*n*_G_), citric acid (*n*_Ci_) and itaconic acid (*n*_It_), and a variable representing the unknown number of moles of water produced (*υ*).

5 *n*_G_ G + *n*_Ci_ Ci  +*n*_It_ It → 1 P(GCiIt) + *υ* H_2_O

The polyesterification reaction can be considered to consist of two possible polymerisation reactions between the triol glycerol and either the triacid citric acid or the diacid itaconic acid. By setting the glycerol stoichiometric variable to unity (*n*_G_ = 1), Fig. 4 shows how the two separate reactions can be considered as if they were stoichiometric ranges, where there exist stoichiometric bounds between which polymerisation is possible.

**Fig. 4:**
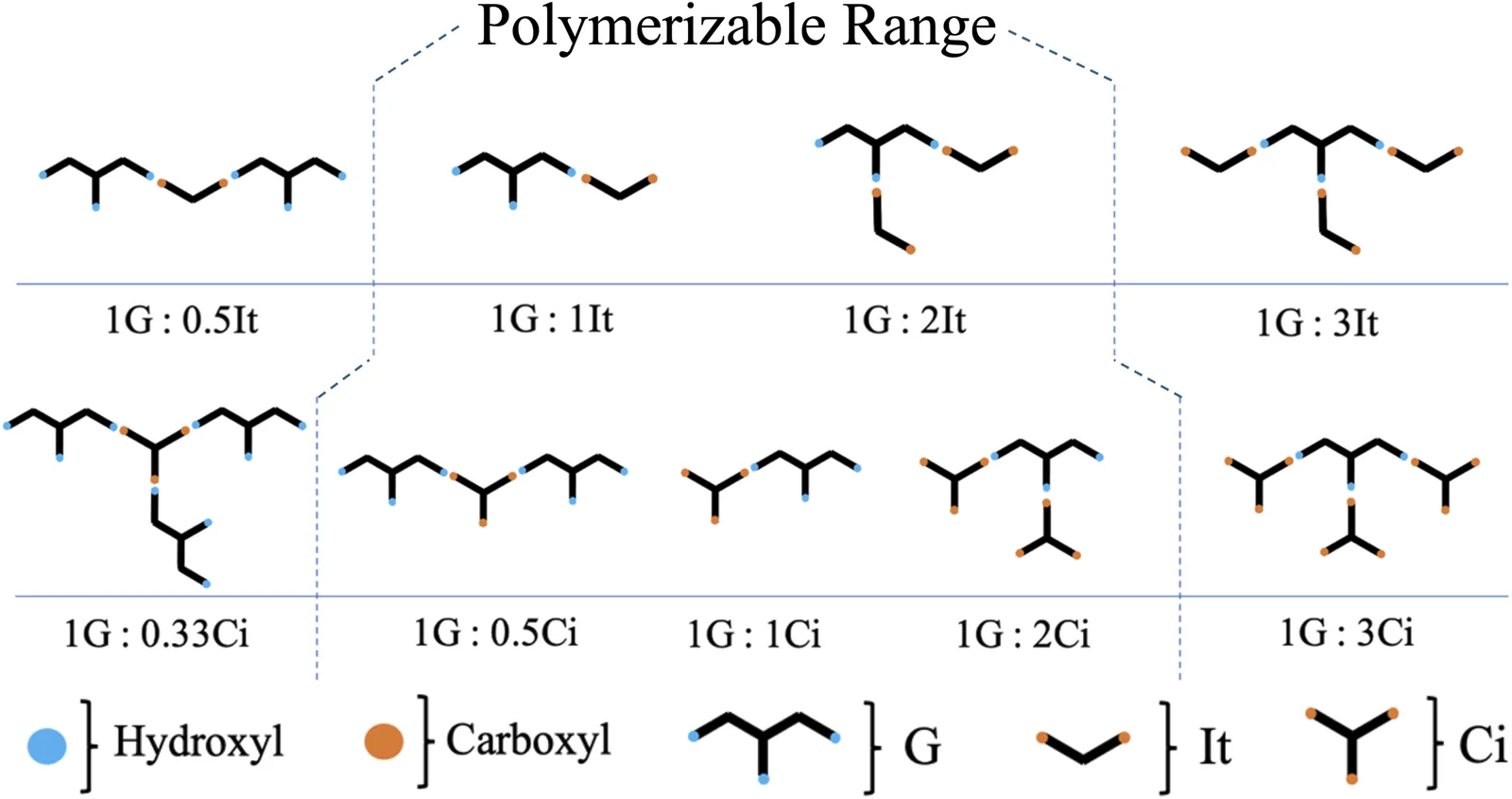
Simplified chemical representations displaying the polymerisable range of the two different triol–acid combinations encountered during poly(glycerol citrate itaconate) synthesis. These ranges represent the stoichiometric intervals where difunctional and trifunctional monomers allow network formation; outside these bounds gelation may not occur. In the legend: “G” glycerol, “It” itaconic acid, “Ci” citric acid.

These stoichiometric bounds are used to directly constrain the stoichiometric variables; eqn (6) describes the lower (*n*^l^_Ci_) and upper (*n*^u^_Ci_) bounds placed on the citric acid stoichiometric variable, whilst eqn (7) shows those for lower (*n*^l^_It_) and upper (*n*^u^_It_) bounds on proportion of itaconic acid.

6 *n*^l^_Ci_ ≤ *n*_Ci_ ≤ *n*^u^_Ci_ = 0.5 ≤ *n*_Ci_ ≤ 2.0

7 *n*^l^_It_ ≤ *n*_It_ ≤ *n*^u^_It_ = 1.0 ≤ *n*_It_ ≤ 2.0

In addition to the basic polymerisable bounds imposed on the two stoichiometric variables, a further constraint must be imposed for a successful optimisation: it must be possible for permutations of prepolymer monomeric mixtures to allow one acid to entirely dominate the other whilst ensuring that a prepolymer will still polymerise – this constraint ensures that any polymerisable stoichiometric mixture of monomers is possible. To achieve this, a three-dimensional bounded domain (a.k.a. parameter space, *A*) was devised consisting of three fundamental predictor variables: firstly, a citric acid strength predictor variable (*s*_1_) influencing the citric acid stoichiometric variable. Secondly, an itaconic acid strength predictor variable (*s*_2_) influencing the itaconic acid stoichiometric variable. Thirdly, a balancing predictor variable (*b*_1_) capable of trading off the two acid systems, influencing both the citric and itaconic acid stoichiometric variables. 
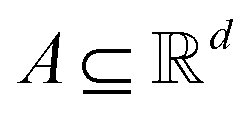
 describes the parameter space *A* as a multi-dimensional real of *d* dimensions, where *d* = 3 given our three predictor variables. All three predictor variables took constrained ranges of between 0 and 1 (*s*_1_ = [0, 1], *s*_2_ = [0, 1], *b*_1_ = [0, 1]), and were linked mathematically to the stoichiometric variables (*via*eqn (8) and (9)).

8 *n*_Ci_ = (*n*^l^_Ci_ + (*n*^u^_Ci_ − *n*^l^_Ci_) × *s*_1_) × *b*_1_

9 *n*_It_ = (*n*^l^_It_ + (*n*^u^_It_ − *n*^l^_It_) × *s*_2_) × (1 − *b*_1_)

In summary, any random set of predictor variable values drawn from their respective constrained ranges of 0 to 1 (*e.g. s*_1_ = 0.75, *s*_2_ = 0.25, *b*_1_ = 0.5) could be used to calculate a unique permutation of stoichiometric variables (*e.g. N*_Ci_ = 0.81, *N*_It_ = 0.63) which, on mixing of a prepolymer in accordance with eqn (10) and curing, would give rise to a unique polymer and associated mass loss (*e.g.* Δ*m*_%_ = 30).

10 *n*_G_ G + 0 ≤ *n*_Ci_ ≤ *n*^u^_Ci_ Ci + 0 ≤ *n*_It_ ≤ *n*^u^_It_ It → 1 P(GCiIt) + *υ* H_2_O

The sequence of calculations linking abstract predictor variables (*s*_1_, *s*_2_, *b*_1_) to the practical stoichiometric variables (*n*_Ci_, *n*_It_) and onwards to the target variable of mass loss (Δ*m*_%_), allows the expression of a black-box mapping between predictors and targets. This black-box mapping is described by 
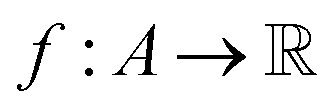
, where an unknown function (*f*) maps any point in our parameter space (*A*) to a single real number value 
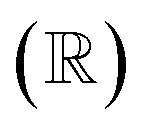
. Eqn (11) more explicitly expresses the black-box function in terms of our target variable and predictor variables at a given sample number (*i*). Note that sample number is an integer counter used to track the number of function evaluations carried out during a sequential optimisation.

11 Δ*m*_%_ = *f*(**x**) = *f*([*s*_1_^*i*^, *s*_2_^*i*^, *b*_1_^*i*^])

Our optimisation problem is thus expressed in eqn (12) as the maximisation of the black-box function, where the global optimum set of predictor variables (x*) is sought.12
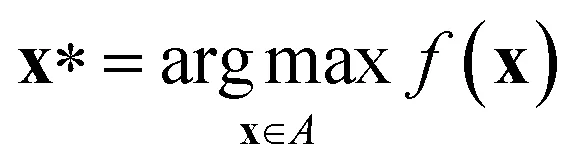


### 2.4 Optimisation algorithm

The sequential optimisation algorithm employed during this work is summarised by the flow diagram in Fig. 5. Successful conclusion of the optimisation is controlled by an if-else clause comparing an initially specified permissible number of function evaluations (a.k.a. sample limit, *p*_tot_) with the current sample number (*i*): when *i* < *p*_tot_ the algorithm continues. A second if-else statement controls whether a dataset exists from which a Gaussian process prior might be built for Bayesian sampling. When available, the algorithm uses the Gaussian process to draw a pre-defined number of samples (*p*_iter_) from the parameter space for evaluation. If the prior-forming dataset is lacking, an initial quasirandom (Sobol) sampling of the parameter space with a pre-defined number of deterministic samples (*p*_init_) is initiated. Samples drawn from the parameter space are processed into stoichiometric variables (*n*_Ci_, *n*_It_), with which an experimenter can manufacture sample(s) before evaluating the target variables Δ*m*_%_ value(s) and returning these to the algorithm. The algorithm seen in Fig. 5 was implemented in the Python programming language, and is incorporated into the ‘BO4BT’ package described earlier.^34^

**Fig. 5:**
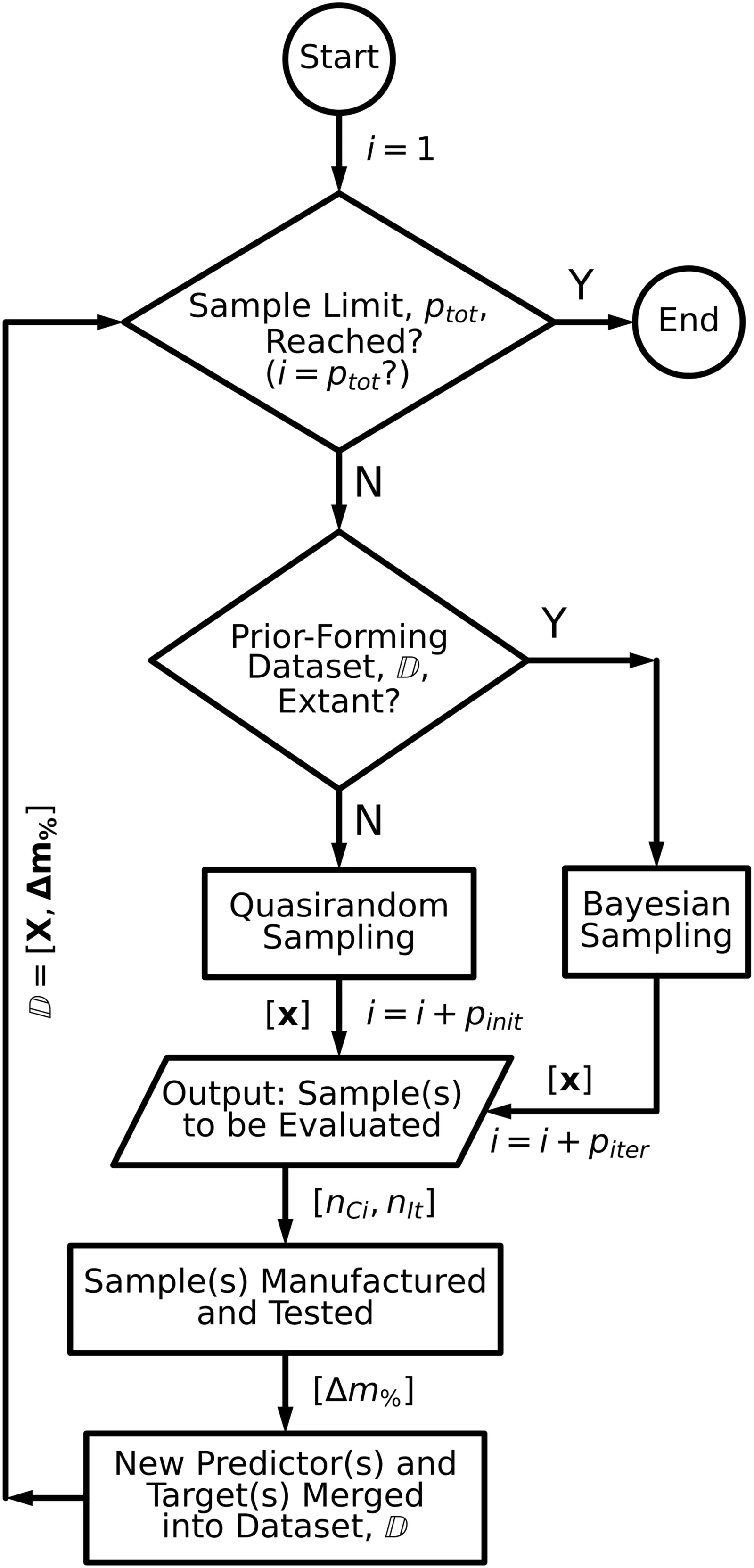
Flow diagram depicting the sequential optimisation algorithm developed.

On calculation of stoichiometric variables during an iteration, the algorithm merges all new sets of predictors variable values into a growing predictor-matrix (**X**), shown in eqn (13).13
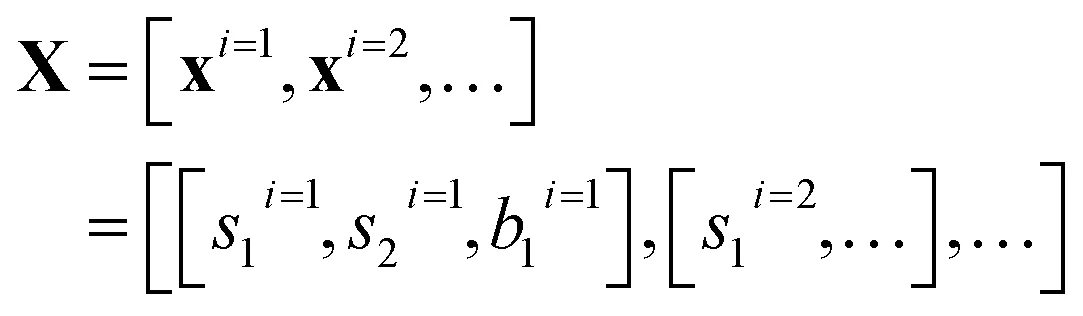


On conclusion of an iteration, the algorithm merges all data associated with samples evaluated into an optimisation-governing dataset. Eqn (14) presents this dataset 
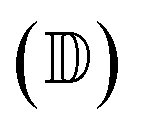
 as a matrix which consists of the predictor-matrix (**X**) and an array of target variable values (**Δm**_%_).14
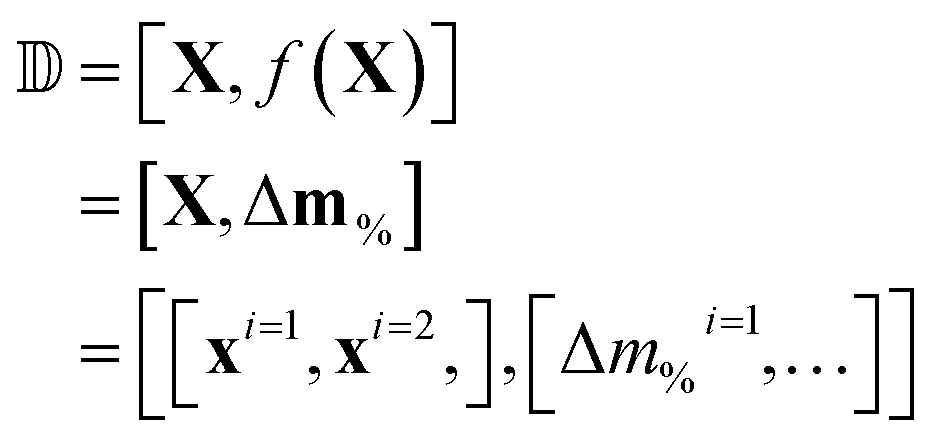


### 2.5 Sampling procedures

During a sequential optimisation two forms of sampling were employed: firstly, a deterministic space-filling sampling of a quasirandom nature was used to representatively sample the parameter space when initialising the optimisation. Subsequently, a Bayesian sampling technique was used to sample-efficiently guide the search for the global optimum x*.

#### 2.5.1 Initial quasirandom sampling

Optimisation routines were initiated by representatively sampling across the parameter space *A*; achieved using the quasirandom Sobol sampling algorithm to draw a preset number (*p*_init_) of samples from across *A*. Sobol sampling was employed as it provides the same or better space-filling properties across 
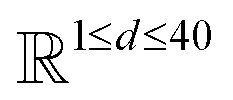
 domains as compared with pseudorandom or grid techniques.^36^ On evaluation of the initial representative set of samples ([**x**^*i*=1^, …, **x**^*i*=*p*_init_^]) for the target variable values ([Δ*m*_%_^*i*=1^, …, Δ*m*_%_^*i*=*p*_init_^]), an initial prior-forming dataset 
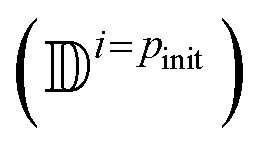
 is produced. In practice, only one quasirandomly sampled initial prior-forming dataset 
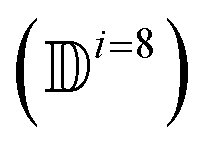
 was generated and used (visualised in Fig. 6). As an aside, a relatively smooth topography witnessed during preliminary experimentation in lower dimensions was the justification for the use of only 8 samples during initial quasirandom sampling.

**Fig. 6:**
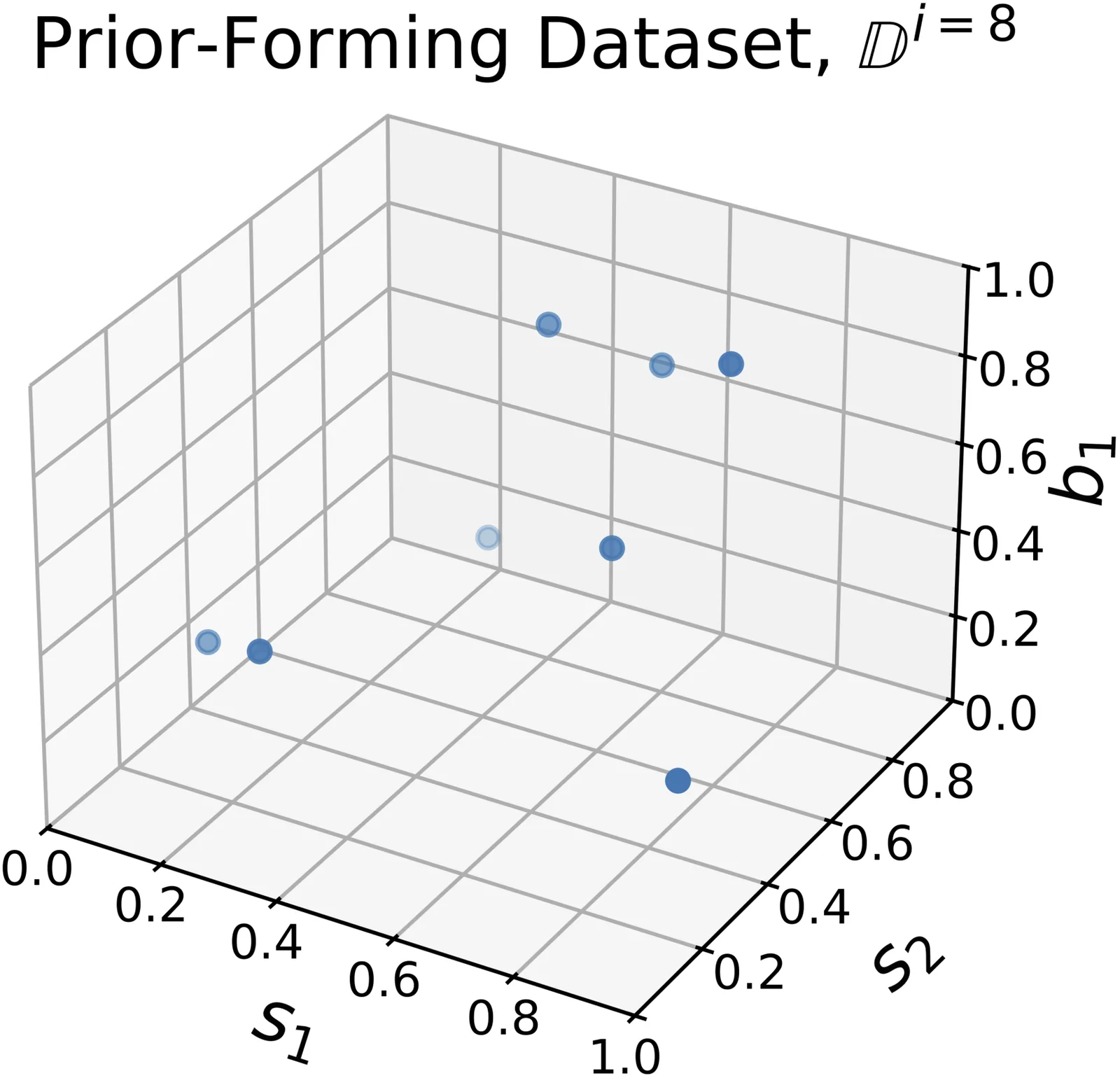
Initial 8 samples quasirandomly from across the parameter space, *A*, comprised of *s*_1_, *s*_2_, and *b*_1_ (as in eqn (8) and (9)), which provide the prior-initialising dataset for the Bayesian routine. The coloration of points denotes distance from viewer with darker points appearing further away.

#### 2.5.2 Sequential Bayesian sampling

Sequential sampling by means of Bayesian optimisation was undertaken using a Gaussian process regression model, which was derived from the regression of an optimisation dataset 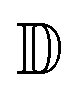. Eqn (15) shows how the Gaussian process
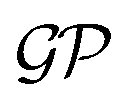
, is a distribution over functions which is completely described by both a mean function, *m*, and a covariance function, *K*.15
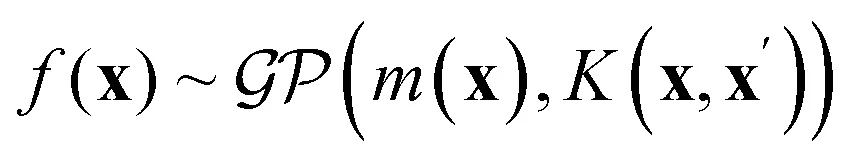


As an infinite-dimensional generalisation of multivariate normal distributions the Gaussian process inherits the properties of a conjugate prior,^37^ allowing the initial posterior (*e.g.*
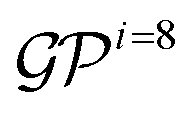
) to be recycled as a prior alongside new data (*e.g.*
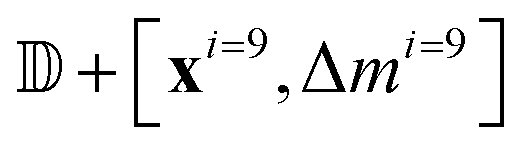
) to generate an updated posterior (*e.g.*
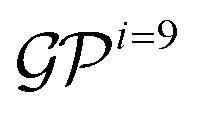
) in accordance with Bayes' theorem. The surrogate model evolves during sequential optimisation as new data are incorporated at each iteration, *i*.

During the fitting of the Gaussian process, a covariance function is used to encode beliefs regarding the families of functions likely to fit the data observed. In this work we used a Matérn covariance function containing a hyperparameter controlling smoothness of functions drawn from the Gaussian process. This was set to *ν* = 2.5, balancing the need for smoothness in sample paths against the drawbacks of higher values of *ν* (*e.g.* unphysical nature of infinitely differentiable functions or mathematical instabilities when undertaking likelihood calculations).

After generating a posterior, a simple one-step lookahead sampling policy was used to select a suitable next point **x** within the parameter space *A* to sample. In this work, we focused on policies making use of the ‘simple reward’ utility function (see eqn (16)) which takes the current dataset 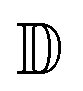, and from the set of sample coordinates evaluated so far *X* returns the predicted mean value Δ*m*_%_ of the point **x** exhibiting the highest predicted mean (max *m*(*X*)) according to the current posterior 
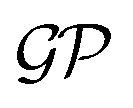
.^38^16
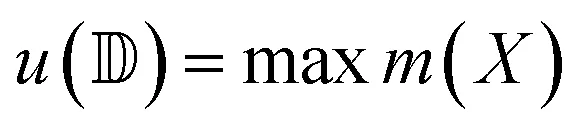


In adopting this utility function, two different sampling policies were developed and investigated. The first was predicated on the ‘probability of improvement’ acquisition function (defined in eqn (17)), where any given putative **x** is scored by the probability that it will produce an updated dataset 
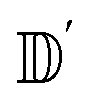
 which generates a higher evaluation from the utility function than some absolute threshold *τ*. The probability of a sample exceeding the current best is deduced using the current posterior 
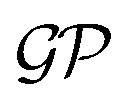
’s mean (*m*(**x**)) and standard deviation 
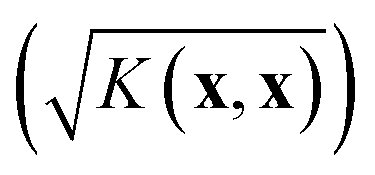
 measured at that sample position. Here, the improvement threshold was defined as the current best observed value 
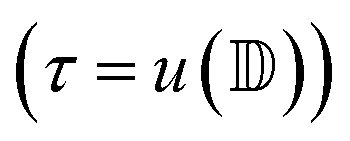
, rather than introducing an additional offset parameter *ε*. This simplified the optimisation while avoiding unnecessary hyperparameter tuning.17
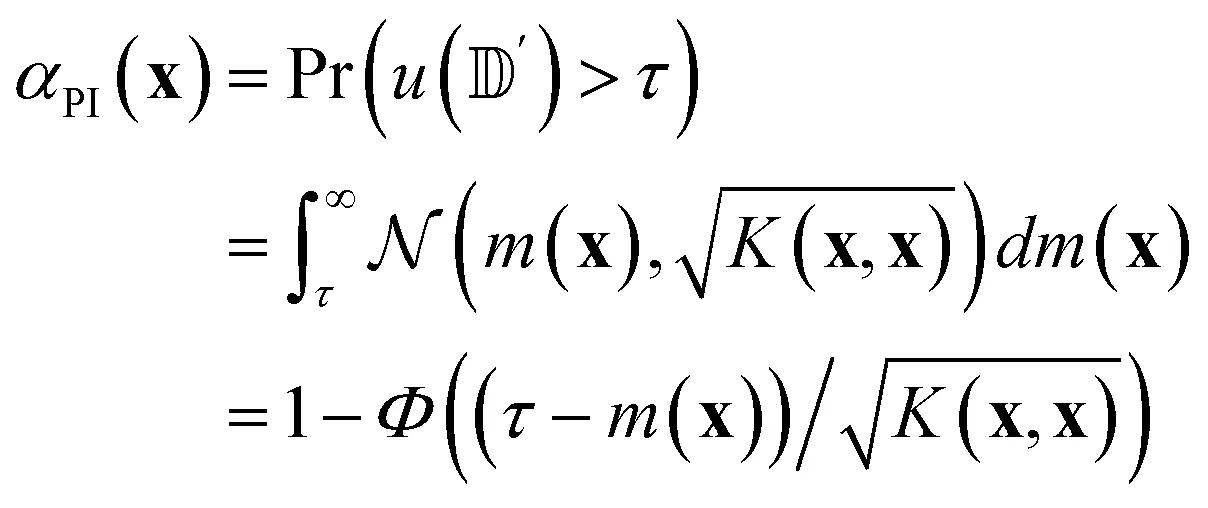


The second policy made use of the ‘expected improvement’ acquisition function (defined in eqn (18)), which incorporates an expectation of improvement value 
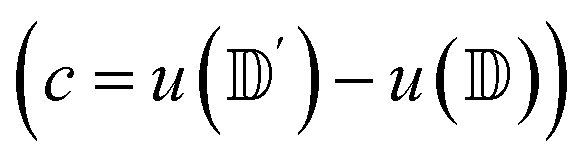
. This counteracts any overtly exploitative behaviour by rewarding both the scale and probability of an improvement above the threshold *τ*.^39^18
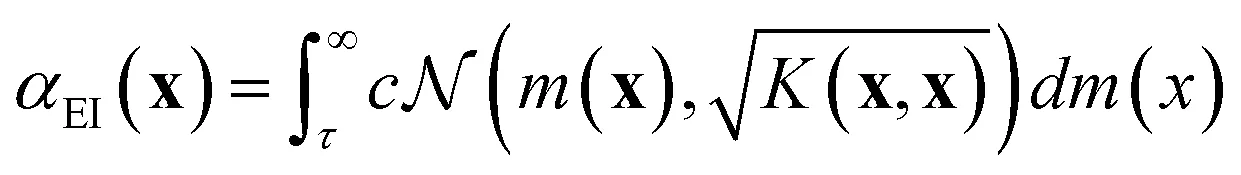


Eqn (19) shows how the next point to be sampled, **x**^+^, is found by trivially optimising the cheap acquisition function (many putative samples **x** may be evaluated) to find the global optimum within the bounds of the parameter space *A*.^39^19
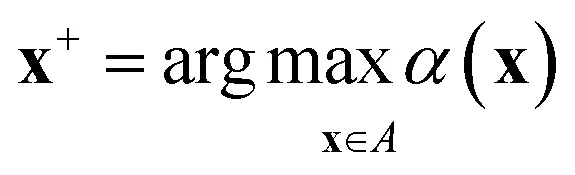


In the present work both of these acquisition functions are employed during computational experiments concerning an emulated parameter space, whilst only probability of improvement was deployed in practice. Gaussian processes and acquisition functions were implemented using two open-source packages: scikit-optimize (“skopt”, v0.9.0)^40^ and Ax (“ax-platform”, v0.4.3).^41^ Note that the Gaussian process regression models developed by these software packages assumed that measured percentage mass loss values comprised both an underlying composition-dependent response plus some independent and normally distributed noise. Both the Ax and scikit-optimize packages inferred the homoscedastic noise during the fitting of the Gaussian process, with the *σ* value being equal to 0.46% points for the scikit-optimize fitted 
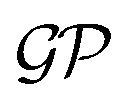
 (at the culmination of the practically-undertaken optimisation routine).

#### 2.5.3 One-shot space-filling sampling

To facilitate the assessment of the Bayesian techniques' sample efficacy, two simple one-shot sampling techniques were employed so that benchmark values could be obtained. This was achieved by using either pseudorandom or quasirandom (Sobol) sampling techniques to disperse the maximum allowable number of samples (*p*_tot_ = 27) across the parameter space *A*. Subsequently, for any technique deployed, all samples, **X**, were evaluated, *f*(**X**), to retrieve associated target variable values, Δ*m*_%_, generating a complete dataset 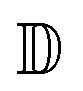. The highest target variable value was identified within each technique's dataset (
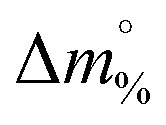
) and equated to a benchmark value. The present work deployed both during computational experiments, whilst only the pseudorandom sampler saw practical application. Fig. 7 visualises the spread of samples it generated, and on functional evaluation of this set the best mass loss discovered was recovered 
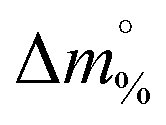
 = 14.01.

**Fig. 7:**
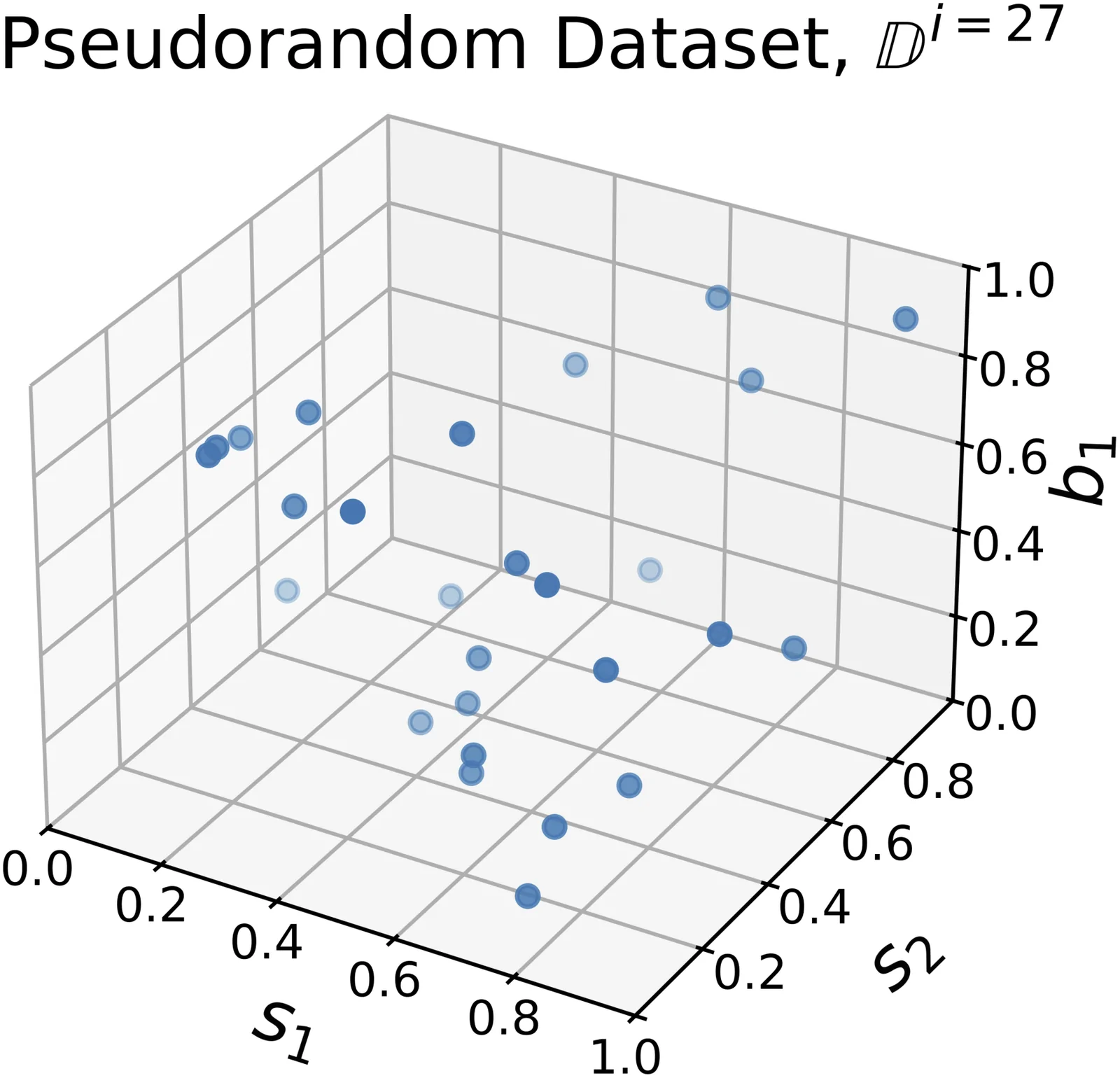
Spread of the 27 samples drawn by the space-filling one-shot pseudorandom sampling technique during practical experimentation. The coloration of points denotes distance from viewer with darker points appearing further away.

#### 2.5.4 Sequential space-filling sampling

To provide a more robust comparator of the Bayesian techniques, a second sequential sampling technique was employed during computational experimentation. Here, we re-constructed a sequential space-filling sampling technique developed by Crombecq *et al.* (2011) called the mc-intersite-proj-th (MIPT) algorithm.^42^ At each iteration during a sequential sampling routine this objective-indifferent technique undertakes a Monte Carlo sampling exercise, wherein each of the vast number of putative candidates have their ability to fill space (within the parameter space and between the samples evaluated so far) quantified and the strongest candidate selected for evaluation.

### 2.6 Emulation of physical system

Since repeated experimental optimisation was impractical, a computational emulator of the physical system was constructed to facilitate statistical comparison of the sampling strategies. In fact, five emulators were developed, four being based on Gaussian processes (with differing covariance functions and hyperparameterisations) whilst the fifth employed gradient boosted decision trees (GBDT). The dataset informing the development of these emulators was comprised of all experimentally evaluated samples from both preliminary investigation of the parameter space, preparation of the pseudorandom benchmark, and the Bayesian optimisation routine employing the probability of improvement acquisition function. This consolidated dataset consisted of 83 stoichiometric variable arrays (*e.g. n*_ci_, *n*_it_) and associated target variable evaluations (*e.g.* Δ*m*_%_). Predictor variable arrays were eschewed as the intent was to train emulators capable of predicting Δ*m*_%_ from *n*_ci_, *n*_it_, reasoning being two-fold: firstly, predictor variable arrays are trivially converted into stoichiometric variable arrays. Secondly, emulators are more easily trained in lower dimensional settings.

Subsequently, the consolidated dataset was split (in the ratio 1 : 2) into both test and train sets. To ensure a suitably representative train set and difficult-to-predict test set, a two step process was undertaken. Firstly, Monte Carlo sampling was used to identify candidate test/train splits which exhibited the least spatial overlap between test and train samples. Secondly, from amongst the highest scoring of candidates, a test/train split was manually selected which displayed both a representative train set spread and a test set which included at least one point in the vicinity of the highest Δ*m*_%_ sample. Fig. 8 provides an overview of the test/train split finally adopted.

**Fig. 8:**
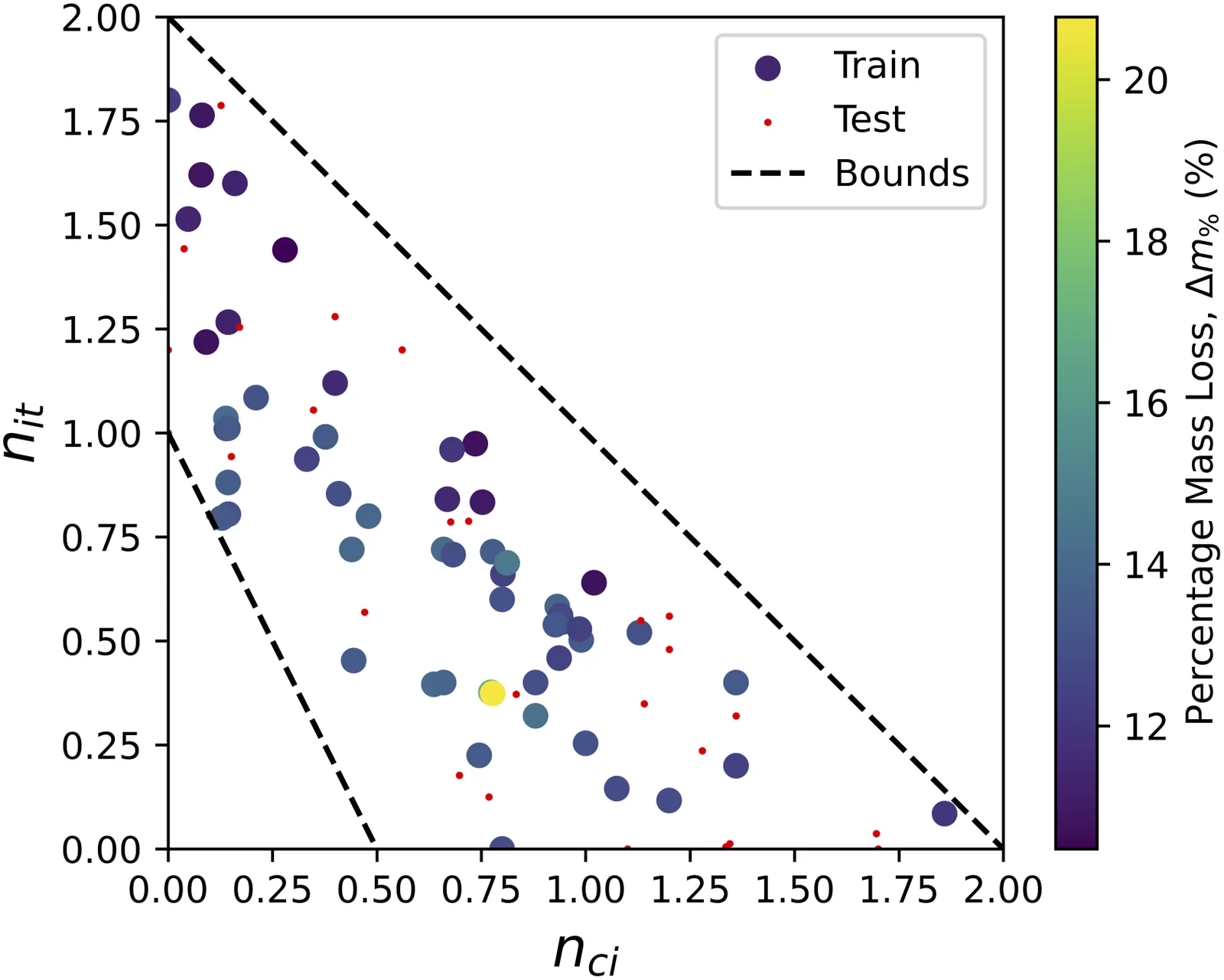
Overview of the test/train split undertaken with respect to the consolidated dataset. Bounds describe the outer edges of the parameter space.

Using the train set, five models were trained: firstly, a series of three Gaussian processes which employed the Matérn kernel were trained, with each of these employing a monotonically increasing value of *ν* = [0.5, 1.5, 2.5] (respectively denoted as “GP-Mat-05”, “GP-Mat-15”, and “GP-Mat-25”). Secondly, a separate Gaussian process was trained which employed the radial basis function as its kernel (denoted “GP-Rbf”). Thirdly, the XGBoost package was used to train a gradient-boosted decision tree model (denoted “Xgb”).^43^Fig. 9 shows a comparison of the five emulators with respect to the functional landscape which they produce when queried over the entire parameter space.

**Fig. 9:**
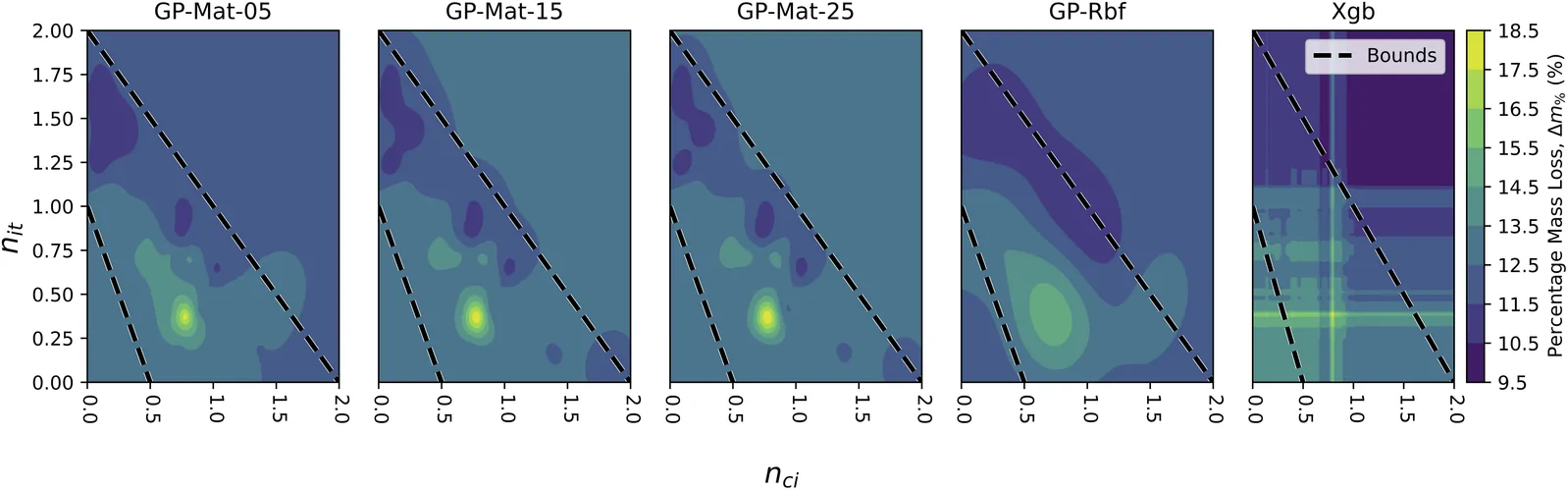
Contour plots describing the functional landscapes across the parameter space as emulated by the five different surrogate models. Bounds describe the outer edges of the parameter space. GBDT: gradient boosted decision tree.

By predicting on each array of stoichiometric variable values from the withheld test dataset, a given emulator could provide a predicted target variable value array. In turn, this array could be used to gauge an emulator's performance by measuring either the mean squared error (MSE) or the root mean squared error (RMSE) between it and the withheld test dataset's array of target variable values. Table 1 presents the performance of the five emulators with respect to these two metrics, as well as providing the emulator's global optimum target variable values. It is clear that although the GP-Rbf emulator displays the strongest performance in terms of RMSE and MSE, it fails to replicate the experimental global optimum value (15.14% < 20.77%); as opposed to the true global optimum associated with the latent objective function, this term denotes the globally optimal result given the experimental data available. Meanwhile, emulator GP-Mat-05 provides both an emulated global optimum approaching the experimental global optimum value (18.57% ≈ 20.77%) whilst retaining similarly low values for both RMSE and MSE, making it a more robust model of the experimental data available.

**Table 1:** Comparison of emulator performance in predicting upon the withheld test dataset. MSE: mean squared error. RMSE: root mean squared error. Max: the emulated global optimum target variable value

Emulator	RMSE (%)	MSE (%^2^)	Max (%)
GP-Mat-05	1.416	2.005	18.57
GP-Mat-15	1.435	2.058	18.27
GP-Mat-25	1.444	2.085	18.29
GP-Rbf	1.414	1.999	15.14
Xgb	1.486	2.209	19.14

## 3 Results

This work produced two sets of results: firstly, those derived from optimisation conducted *via* sampling directly from the latent objective function described by the physical system and secondly, those stemming from the optimisation of the five surrogate functions trained to emulate the latent objective function and the associated physical system. The latter set of results were generated to provide statistical support for the former.

### 3.1 Physical system optimisation

A single Bayesian sequential optimisation experiment was run, with a sample limit (*p*_tot_ = 27), number of samples drawn per sequential iteration (*p*_iter_ = 3), and a prior-forming dataset of 8 quasirandomly sampled targets (Fig. 6). This Bayesian routine employed the probability of improvement acquisition function (eqn (17)). Fig. 10 illustrates the progression of this sequential routine when compared with the one-shot pseudorandom sampling technique, with the mass losses plotted on the *y*-axis being the best values discovered given the samples evaluated so far (
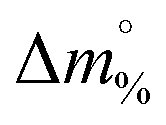
). A simple statistical review of the experimental datasets generated by both the sequential Bayesian and one-shot pseudorandom techniques within the sample limit is provided in Table 2. Given the sequential samples available (*p*_tot_ − *p*_init_ = 19) and the samples per iteration (*p*_iter_ = 3), an extra iteration of sequential sampling was run which consequently breached the sample limit-these extra samples are included in Fig. 10, but are not considered in the preparation of Table 2 (as it would be unfair to compare two different sampling techniques which were allowed an unequal number of samples over which to optimise).

**Fig. 10:**
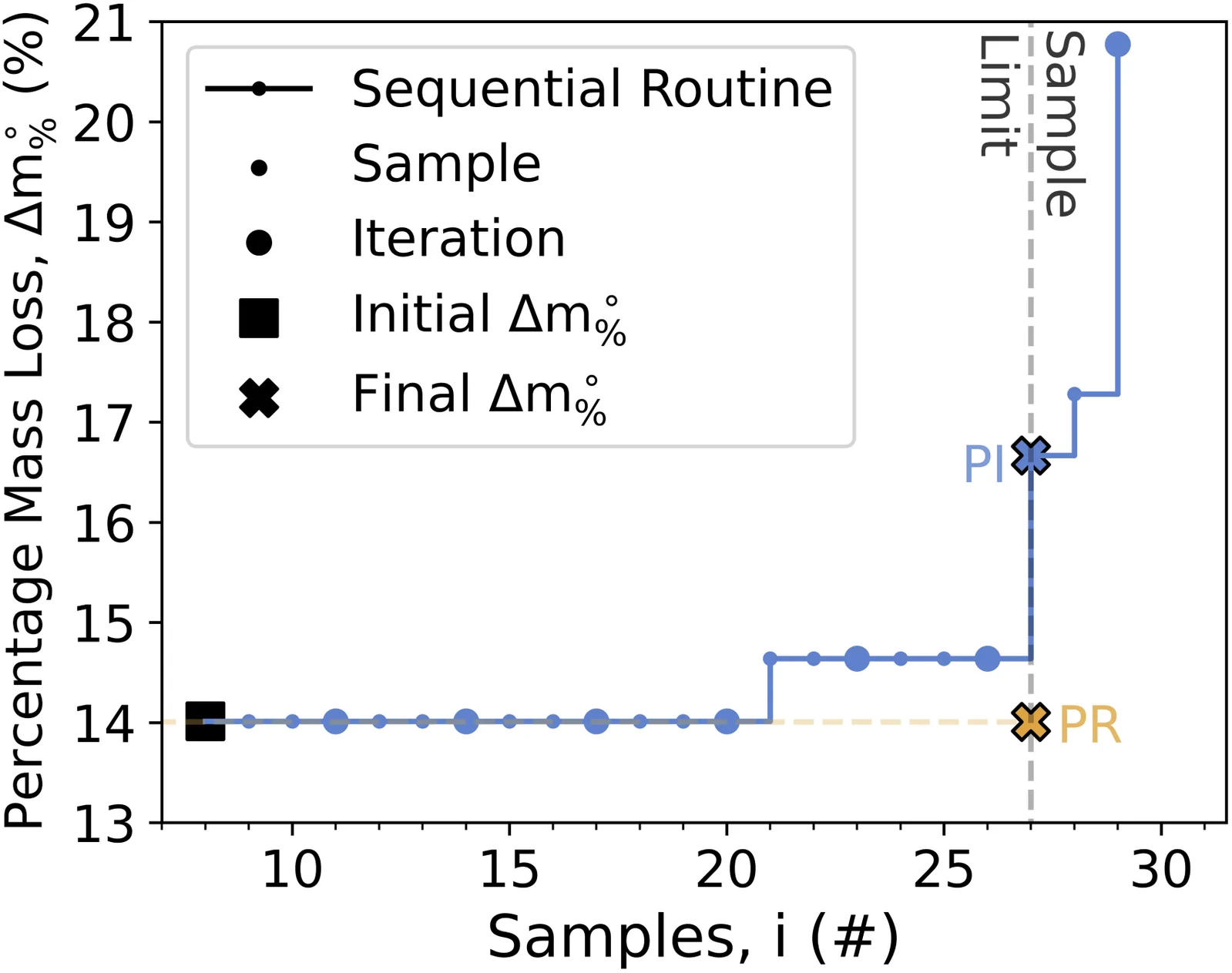
Plot of optimisation progression for the experimentally deployed Bayesian optimisation routine utilising the probability of improvement (PI, solid blue line) acquisition function, and compared alongside the one-shot pseudorandom sampling technique (PR, dashed line). Uncertainty in Δ*m*_%_ values is equal to 0.44% points (1*σ*).

**Table 2:** Statistical review of datasets generated experimentally within the sample limit. P: prior dataset, quasirandomly sampled and fed into Bayesian routine. PI: final dataset from Bayesian routine using probability of improvement acquisition function. PR: dataset from one-shot pseudorandom sampler. No.: number of samples in dataset. Max: highest discovered mass loss (
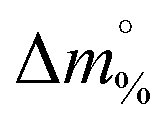
) mean: population mean. Standard dev.: a single standard deviation

Technique function	No.	Max (%)	Mean (%)	Standard Dev. (%)
P	8	14.01	12.82	1.04
PI	27	16.67	12.50	1.63
PR	27	14.01	12.54	0.88

Fig. 10 shows that although the sequential Bayesian technique discovers a sample surpassing the pseudorandom benchmark by sample 21 (77% of *p*_tot_), its first improvement exceeding the estimated uncertainty of the two samples was recorded on sample 27 (100% of *p*_tot_) – this was consistent with exploratory-biased behaviour. Further evidence of the probability of improvement acquisition functions exploratory-biased behaviour is seen in Table 2, where, despite an improvement upon the initial prior's highest discovered target value (14.01% points → 16.67% points), the running mean of samples within the growing dataset fell from its quasirandomly sampled prior's high (12.82% points) to its final lowpoint (12.50% points) which reached an approximate parity with the pseudorandomly sampled benchmark dataset (12.54% points). Contrary to our initial expectations, the evidence presented above shows that the probability of improvement acquisition function does not exhibit overly exploitative behaviour due to the lack of a marginal threshold parameter *ε* being manually tuned.

Notably, the Bayesian sequential technique employing the probability of improvement acquisition function discovered samples attaining the highest mass losses of any sample characterised during this work (
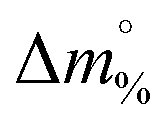
 = 20.8%), albeit outside the sample limit (see Fig. 10).

### 3.2 Emulated system optimisation

Since curing of a single prepolymer sample could take up to a week to complete, the temporal expense of sampling the objective function was too high to allow for repeat experiments involving the sequential techniques deployed. This precluded statistically proving the utility of Bayesian optimisation in this field experimentally within reasonable timeframes. However, by developing inexpensive surrogate functions capable of emulating the physical system, a much larger number of function evaluations could be carried out computationally, facilitating statistical analysis of sampling methods' efficacy. By despatching 1000 simulations of any given sampling technique's ability to optimise any given emulator's surrogate function and returning an array of maximum target variable values 
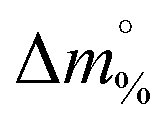
 discovered, basic statistical conclusions could be drawn. All simulated sampling techniques observed a sample limit of *p*_tot_ = 27, whilst the sequential techniques were all initiated using *p*_init_ = 8 quasirandomly drawn samples. Sampling techniques spanned one-shot and sequential forms, with the former group comprised of pseudorandom (PR) and quasirandom sampling (QR) whilst the latter was made up of both objective-naive and Bayesian methods. The lone objective-naive space-filling technique simulated was the mc-intersite-proj-th algorithm (MIPT), whilst the two Bayesian techniques diverged in terms of the acquisition function employed - either probability of improvement (PI) or expected improvement (EI).

Fig. 11 presents a series of strip plots showing distribution of outcomes for the 25 000 simulations undertaken across the five sampling techniques and five emulators studied. On comparison of the mean 
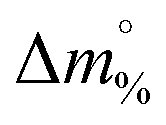
 values discovered by the various sampling techniques, sequential Bayesian techniques were shown to generally perform better (as observed in 4/5 emulators trialled) than either the one-shot or objective naive sequential techniques. Although standard deviation values associated with these mean values would preclude a straightforward conclusion that the difference between values are statistically significant, it can be seen that the simulations are distributed in a somewhat right-skewed fashion which shows that only the Bayesian techniques are capable of discovering well-above-average-performing samples (causing the majority of skew seen in the means).

**Fig. 11:**
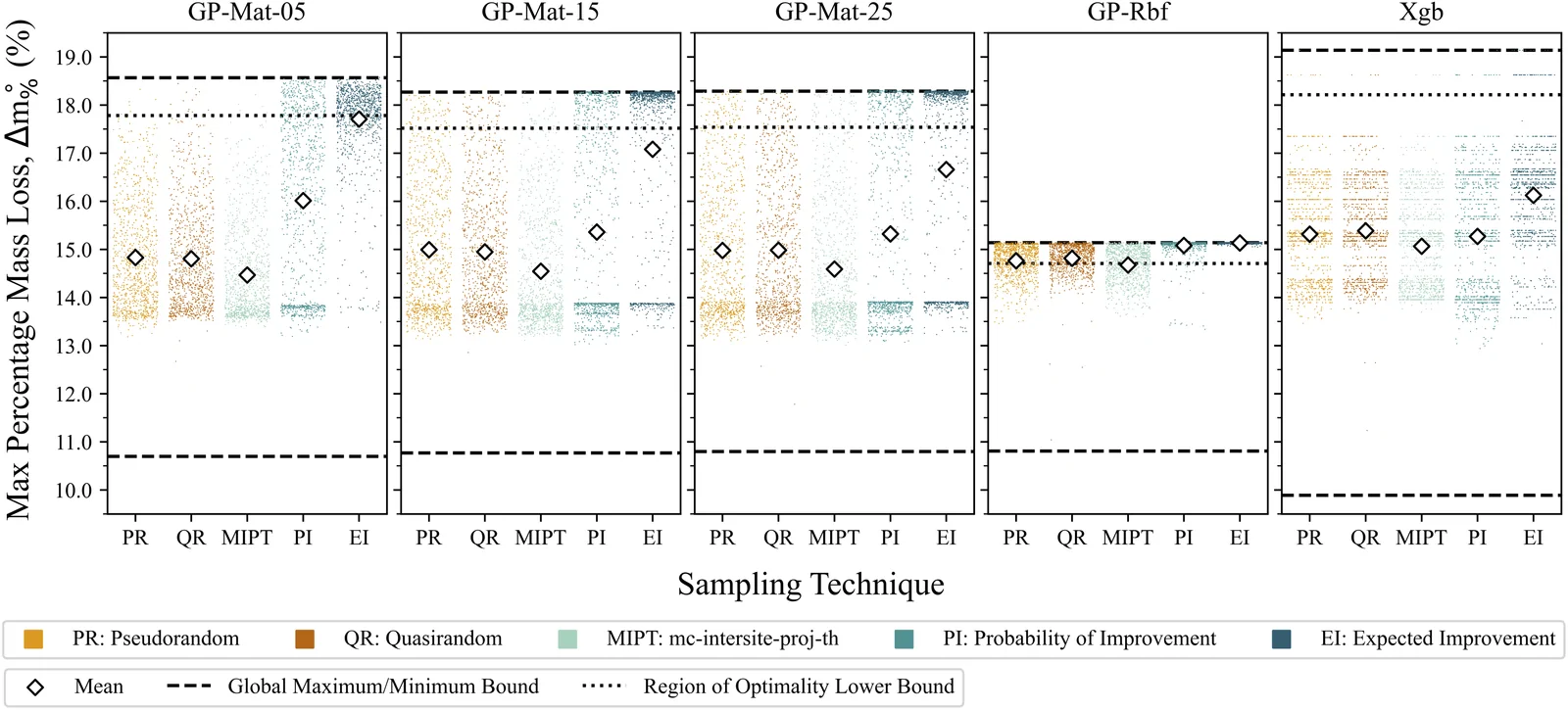
Strip plots of maximum function evaluations obtained 
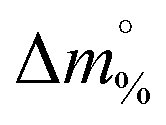
, for each of the 25 000 simulated sampling exercises undertaken. Every simulated sampling exercise observed a constant sample limit *p*_tot_ = 27, with each sampling technique being simulated 1000 times upon each of the emulators, and the Bayesian routines were initiated with 8 quasirandomly drawn samples.

To investigate the degree to which exceptionally high value formulations were uncovered by the various sampling techniques, a ‘region of global optimality’ was devised for each of the emulators, with an upper bound defined by the computational global maximum and a lower bound defined as being 10% lower than this (as relativised by the computational global minimum). The Bayesian sequential technique adopting the expected improvement acquisition function in its sampling policy not only outperformed the probability of improvement technique in terms of mean values attained, but also in number of simulations obtaining 
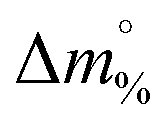
 values which resided within the small region of global optimality, as evidenced in Table 3. Notably, neither of the one-shot techniques nor the objective-naive sequential technique were able to produce as high a proportion of samples within the ‘region of global optimality’, with all three consistently producing either one or two orders of magnitude less samples than that generated by the Bayesian techniques (Table 3).

**Table 3:** Summary of percent of simulations surpassing the lower region of optimality bound. Emu.: Emulators simulated upon. MO5/M15/M25: Gaussian process emulators where *ν* = 0.5/1.5/2.5 respectively. Rbf: Gaussian process emulator with RBF kernel. Xgb: XGBoost gradient boosted decision tree emulator

Emu.	PR (%)	QR (%)	MIPT (%)	PI (%)	EI (%)
M05	0.6	1.3	0.5	23.8	65.0
M15	6.9	5.9	3.4	26.3	69.9
M25	9.1	6.9	5.3	26.6	59.3
Rbf	65.2	71.1	53.0	96.8	100.0
Xgb	0.8	0.4	0.4	1.3	5.1

Taking the GP-Mat-05 as the most representative of the emulators, and subject to the assumptions that it adequately represents the underlying objective function and that the defined ‘region of global optimality’ is a reasonable proxy for the experimentally identified optimum, the estimated discovery probabilities were 65% for expected improvement and 23.8% for probability of improvement. This would make the chances of having discovered these exceptionally well-performing formulations during the course of our one-off implementation of the Bayesian sequential routine significantly more likely than the 0.6%, 1.3%, and 0.5% probabilities exhibited by the two one-shot techniques (PR and QR) or the objective-naive MIPT technique respectively.

## 4 Conclusion

Having engaged in the practical optimisation of the monomeric composition of a simple bio-based branching thermoset polyester (poly (glycerol citrate itaconate)) with respect to an objective of interest (percentage mass loss *via* curing) we have discovered an experimental global optimum of 
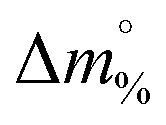
 = 20.77% at the sample coordinates (*s*_1_ = 0.337, *s*_2_ = 0.648, *b*_1_ = 0.773) corresponding to a stoichiometric balance of 1 glycerol to 0.77 citric acid to 0.37 itaconic acid. This was discovered by a sequential Bayesian technique whose sampling policy adopted the well known probability of improvement acquisition function.

The utility of Bayesian techniques in sample-efficient global optimisation was demonstrated through 10 000 simulations of their behaviour across five different surrogate functions emulating the objective function. In the case of the emulator GP-Mat-05, the expected improvement and probability of improvement sequential techniques were found to exhibit a 65.0% and 23.8% probability of uncovering the computational ‘region of global optimality’, whist both the pseudorandom, quasirandom, and objective-naive sequential techniques displayed much lower probabilities (0.6%, 1.3%, and 0.5% respectively).

If the intention is for poly(glycerol citrate itaconate) to perform the role of a useful thermoset resin for moulding applications, future work would have to focus on the mitigation of the bubbling effect, improvement of mechanical strength, and reductions in the cure time. The last of these is of particular importance given the relatively higher amounts of process-energy required to prepolymerise and cure the presently described branched thermosets when compared with conventional unsaturated polyester resins (UPR). Modelling of these two processes with error propagation as described in the supporting information (see Section 8.2) suggested that conventional UPR thermosets require 81% less process energy than the branched-polyester route, with a 5th–95th percentile uncertainty range of 57–91%. This difference was found to be dominated by the prolonged 90 °C oven residence time required for the branched polyester. The estimates remained sensitive to oven power, batch loading, cure duration and the process boundaries applied to prepolymer manufacture. We therefore suggest that future work focuses upon dual-objective optimisation problems which consider both mechanical strength maximisation alongside oven residence time minimisation, whilst adopting predictor variables such as prepolymer stoichiometry, temperature and pressure, and quantities of catalytic reagent added.

Alternatively, different bio-based and readily-available monomers could be considered (*e.g.* succinic acid) which may encourage development of novel thermoset networks that might impact upon the objectives of interest. Note that addition of further monomers to the stoichiometric balancing mechanism described in this paper would, due to the curse of dimensionality, require many more samples to effectively explore and optimise these systems: this would encourage the replacement of human-in-the-loop experimentation with laboratory automation.

## Conflicts of interest

There are no conflicts to declare.

## Supplementary Material

SU-OLF-D6SU00235H-s001

## Data Availability

Custom software developed for the purpose of execution of optimisation routines is available under the ‘BO4BT’ project lodged in GitHub at the following address: https://github.com/ThomasDodd97/BO4BT. The LaTeX project associated with this paper has been made available under the ‘UoC_Paper1’ project lodged in GitHub at the following address: https://github.com/ThomasDodd97/UoC_Paper1. Furthermore, the ‘sandbox’ directory within the ‘UoC_Paper1’ project contains both the data and the Jupyter notebooks required to process it into the graphics depicted in the present work. Supplementary information (SI): details of the Monte Carlo uncertainty-propagation analysis for the mass-loss metric and the process-energy modelling comparing the branched-polyester and conventional UPR routes. See DOI: https://doi.org/10.1039/d6su00235h.
