# Supplementary material for: Bayesian optimisation of a bio-based biodegradable polyester thermoset
Source: RSC Sustain. 2026 Sep 29. Online ahead of print. doi: 10.1039/d6su00235h (PMC13659735; doi:10.1039/d6su00235h)
Supplement: Supplementary file 1 [file SU-OLF-D6SU00235H-s001.pdf]

Supplementary Information (SI) for RSC Sustainability.
This journal is © The Royal Society of Chemistry

Supplementary Information

Thomas J Højlund-Dodd, James Elliott

September 2026

S1 - Notes on Measurement Error Propagation

To evaluate uncertainty associated with the percentage mass loss metric we undertook some error propagation analysis by modelling the process of obtaining values for this metric. This modelling was carried out in the Python programming language and is detailed in a Jupyter notebook held within the Github repository associated with this paper, it can be found under the 'Sandbox/UncertaintyProgagation' directory and is entitled 'UncertaintyModel.ipynb'.

By setting the initial mass of a given sample at 3 g and allowing the final mass to vary in accordance with a uniform distribution between a lower and upper bound (as deduced from the two samples evaluated exhibiting the maximal and minimal mass losses across all trials) we could simulate the drawing of a random sample from across the parameter space each having its own unique mass loss. Additionally, to model the measurement error which manifests itself during the process of evaluating the initial mass and final mass values we could employ normal distributions with standard deviations equal to the repeatability of the scales employed (0.01 g). By coupling these concepts together and undertaking a Monte Carlo sampling exercise we could obtain estimates of the average uncertainty associated with any given percentage mass loss value considered in the present work; this was equal to 0.44 % points (1σ=0.01% points).

S2 - Notes on Process-Energy Calculations

Although this paper has focussed upon branching polyester thermosets as an alternative to unsaturated polyester resin derived thermosets on account of the relative ease with which these materials may be prepared from bio-based feedstocks, we acknowledge the fact that this does not inherently make this family of polymer more sustainable than their conventional counterparts. To prove this, one would first have to choose a suitable quantitative definition of sustainability (e.g. embodied carbon), before engaging in a comprehensive life-cycle analysis for both the methods by which thermosets were manufactured.

Although by no means a full life-cycle analysis of either route, we have undertaken some modelling of the two system with a view to elucidating whether the prolonged thermal cure time used for branched poly(glycerol citrate itaconate) thermosets is likely to impose an energy penalty relative to the unsaturated polyester resin route. This model is presented within a Jupyter notebook file entitled 'calcs_restructured_uncertainty.ipynb' (within the 'Sandbox/EnergyCalculations' directory) in the GitHub repository associated with this paper.

Here, we establish two separate models, one for the branched system and the other for the unsaturated polyester system. These models both consider the energy required to form prepolymers from feedstocks and the subsequent energy required to cure these

prepolymers. Each model is based on a set of parameters, each parameter being described by a triangular distribution, which allows propagation of uncertainty to be considered over the course of a large number of simulations in a Monte Carlo fashion.

Metric	5th Percentile	Median	95th Percentile
Branched Polyester Route (kWh kg^{-1})	24.4	49.9	84.1
Conventional UPR Route (kWh kg^{-1})	5.4	9.2	14.6
UPR/Branched Ratio	0.087	0.186	0.431

Table 1: *Summary of process-energy modelling exercise.*

On conclusion of 20,000 Monte Carlo draws, it was concluded that the more energy intensive route was that of branched polyester thermoset manufacture (requiring an average of 5.4× the kWh kg^{-1} of thermoset). Table 1 provides an overview of this exercise, and shows the progagated uncertainty bounds surrounding the median values. Estimates of kWh kg^{-1} yielded by the branched model were particularly sensitive to power required to maintain the oven temperature as well as the cure duration, whilst the unsaturated polyester model was most sensitive toward energy required to maintain reactor temperature, auxiliary power requirements, and heat capacity of various elements.
